# Measurement of Soot Concentration in Burner Diffusion Flames through Emission Spectroscopy with Particle Swarm Optimization

**DOI:** 10.3390/s24041292

**Published:** 2024-02-17

**Authors:** Zizhen Li, Ni Wan, Xiangchen Qian

**Affiliations:** School of Control and Computer Engineering, North China Electric Power University, Beijing 102206, China; lizizhen0203@163.com (Z.L.); wn_201509@163.com (N.W.)

**Keywords:** soot concentration, emission spectroscopy, burner flame, thermocouple particle density method

## Abstract

Measuring soot concentration in a burner flame is essential for an in-depth understanding of the formation mechanism and to abate its generation. This paper presents an improved emission spectroscopy (ES) method that uses an adaptive particle swarm optimization (APSO) algorithm for measuring the concentration of soot in methane burner flames. Experimental tests were conducted on a laboratory-scale facility under a methane flowrate ranging between 0.6 and 0.9 L/min. A comparison analysis of the soot concentration measured by the ES method, the improved emission spectroscopy (IES) method, and the thermocouple particle density (TPD) method (as a reference) was conducted. The ES method obtained a maximum absolute deviation of 0.84 ppm from the average soot concentration at the three measurement points compared to the TPD method, while that of the IES was only 0.09 ppm. The experimental results demonstrate that the proposed IES method can obtain a more accurate soot concentration of diffusion flames.

## 1. Introduction

The primary environmental pollutant emission is from the combustion process of hydrocarbon fuels, such as coal, petroleum, and natural gas [1]. The combustion of natural gas generates fewer emissions of air pollutants and CO_2_ compared to other types of hydrocarbon fuels [2]. Therefore, using natural gas for power generation to replace coal and petroleum has become a prospect. However, as the main component of solid pollutants generated while burning [3], soot contributes to the greenhouse effect and acid rain, and severely reduces atmospheric visibility [4]. 

Soot is formed during the incomplete combustion of hydrocarbon fuels (methane, ethylene, propane), and the formation mechanism is very complex. In recent years, with the increasing attention paid to pollutant emissions and environmental issues, considerable research has been conducted on the formation mechanism of soot. Numerical methods for simulating soot and intermediate components have also been developed [5,6,7,8]. However, under certain operating conditions, the simulation of soot generation is still unable to provide satisfactory results. The main reasons include the lack of an accurate and non-invasive measurement of the physical and chemical properties of soot particles during their evolution in the combustion chamber. Moreover, computational limitations exist in software simulations of molecular weight growth in large gas-phase species, where nucleation of these speciation particles, detailed chemical processes of particle growth, chemical and physical evolution, and oxidation under combustion conditions are difficult to model accurately. Therefore, the chemical pathways involved in soot generation with different flow fields and burner characteristics still need to be studied. The contact measurement method is the primary method for soot measurement, such as the thermocouple particle density (TPD) method [9]. In order to obtain the soot concentration, Lu et al. [10] inserted a thermocouple into a flame and analyzed the influence of gradually accumulating soot particles on the surface of the thermocouple based on the output voltage signal. However, the contact measurement method can only obtain the soot concentration at a fixed point using a relatively long measurement time, and the insertion of the thermocouples causes interference with the flame. 

The flame combustion process is dominated by radiation heat transfer, and the radiation spectrum of the flame contains useful information related to the combustion process. Therefore, the optical measurement methods for soot concentration in combustion flames have significantly progressed in recent years [11,12,13,14]. Emission spectroscopy (ES) has been widely studied and applied because of its simplicity and low equipment requirements. Ai et al. [15] innovatively combined ES with the trichromatic method and realized the two-dimensional visualization of symmetric flame temperature and soot concentration distribution. Lou et al. [16] took two monochromatic images of an ethylene flame at different wavelengths with a charge coupled device (CCD) camera. The obtained two-dimensional images were used to determine the temperature and soot concentration distributions by decoupling reconstruction. However, the ES method has also encountered challenges affecting measurement accuracy. Firstly, it approximates the flame as a grey body and uses the same emissivity in various bands for parameter calculation, but the actual flame does not conform to the ideal grey body radiation characteristics. Secondly, in order to simplify the calculation, the reconstruction model often ignores the influence of the flame self-absorption term and assumes the complex combustion flame as an optically thin flame. For the flame with a small optical thickness, the neglect of the self-absorption term has little influence on the reconstruction accuracy. However, in a flame with large optical thickness, the neglect of the self-absorption term will cause considerable reconstruction errors. Regarding the reconstruction algorithms, in order to retrieve the flame temperature and soot concentration from the measured flame spectral information, the optimization of the algorithm is a key point. Existing research has focused mainly on using algorithms such as Abel inversion and Tikhonov regularization [17,18]. In recent years, some scholars have also used global optimization algorithms, such as particle swarm optimization (PSO), to solve this problem [19,20]. PSO is a population-based heuristic search technique in which each particle represents a solution within the search space. The standard PSO algorithm converges rapidly during the initial stage of a search, but it gradually becomes steady considerably and can become trapped in local optima. Therefore, it is necessary to conduct in-depth research and improvement on the flame radiation method.

This paper improves the ES-based soot concentration reconstruction method using the adaptive particle swarm optimization (APSO) algorithm. Validation tests were conducted under a variety of typical experimental conditions. This study improved the standard PSO by reducing swarm size and the number of iterations to achieve faster reconstruction, thus making the algorithm more efficient with less computational effort. Comparative analysis with the standard PSO algorithm suggests that the new algorithm effectively reduces the error caused by self-absorption, and the average soot concentration is more consistent with the measurement results of the TPD method.

## 2. Image Processing Principles

### The Improved Emission Spectroscopy Method with Particle Swarm Optimization

**Figure 1:**
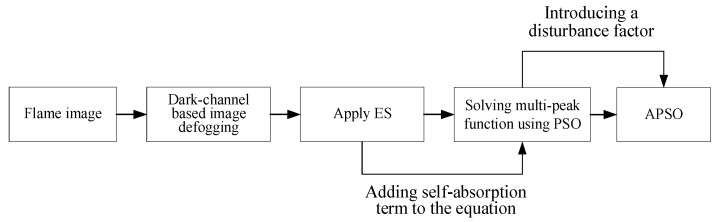
Flow chart of the proposed IES method.

**Figure 2:**
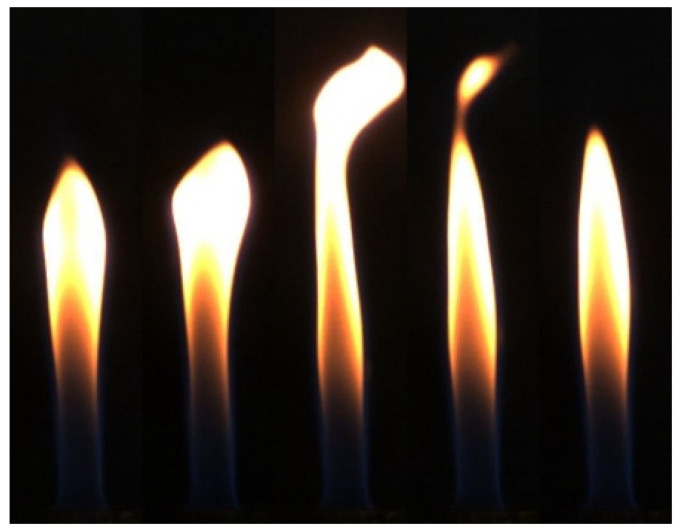
Flame image taken under laboratory conditions (working conditions: methane 0.7 L/min diffusion flame).

The histogram equalization and dark-channel-based image defogging algorithm are used to enhance the contrast of the flame images. The overall experimental process is shown in Figure 1. The dark-channel-based image defogging algorithm is used to improve image contrast [21]. In a flame image, some pixels will always have at least one color channel with the lowest light intensity value, so the minimum value of each light intensity component in each pixel’s red, green, and blue channels can be converted to a grey scale [22]. Then, a minimum value filter is applied to the resulting greyscale image, which in turn results in a dark channel image. For a flame image, the dark-channel-based defogging algorithm is able to eliminate the scattered light present in the background of the image (as shown in Figure 2), which can be applied to temperature calculations.

**Figure 3:**
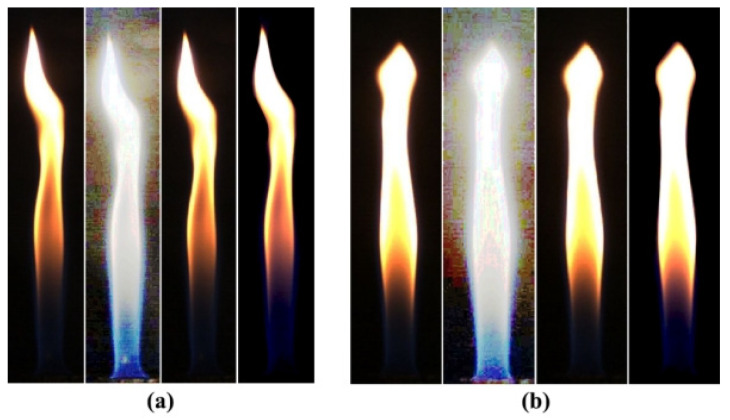
The original, RGB, HSI, and dark-channel defogged images of the flame under methane flowrates of (**a**) 0.7 L/min and (**b**) 0.8 L/min.

As can be seen from Figure 3, the histogram equalization method in RGB (Red, Green, Blue) space brings severe distortions to the original image because the probability density of the grey image scale is uniformly distributed after the transformation in RGB space. For flame images that have a significant contrast with the background, the calculation will result in severe distortions due to greyscale homogenization [23]. Histogram equalization in HSI (Hue, Saturation, Intensity) space is better adjusted than RGB space, with higher contrast improvement. 

**Figure 4:**
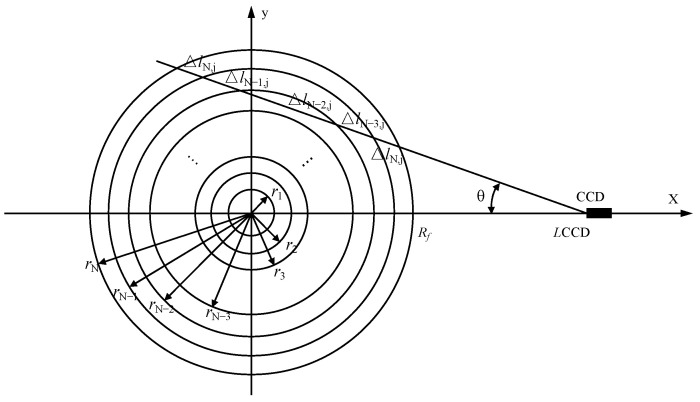
Schematic diagram of the ES method.

Flame radiation refers to the radiation characteristics of gas and solid particles in the flame generated by the combustion of fuel. The ES method is based on the fact that the expression for the intensity of line-of-sight radiation from an optically thin flame is the path integral of its characteristic field, as shown in Figure 4. By dividing the flame into circles along the radial direction and assuming that the soot concentration and flame temperature do not change in each circle, the monochromatic radiation intensity embodied in the flame image is the sum of the monochromatic radiation intensity distributions of each point in the direction of a CCD line of sight [24]. Therefore, a one-dimensional tomographic reconstruction of the characteristic field of the flame can be achieved by external scanning along the flame in different locations.

(1) $$I\left(\lambda \right)=\int \kappa_{\lambda }\left(x,y\right)I_{b\lambda }\left[T\left(x,y\right)\right]\exp\left[-\int_{\left(x,y\right)}^{\infty }\alpha_{\lambda }\left(x^{\prime },y^{\prime }\right)ds^{\prime }\right]ds$$

Equation (1) integrates the radiation intensity along the radiation path length, where I(λ) is the overall line-of-sight radiation intensity and $I_{B\lambda }(T)$ is the Planck function:

(2) $$I_{B\lambda }\left(T\right)=\frac{C_{1}}{\lambda^{5}\left(\exp\left(\frac{C_{2}}{\lambda T}\right)-1\right)\pi }$$

where *T* is the flame temperature, *C*_1_ and *C*_2_ are the first and second Planck radiation constants, respectively, and $\kappa_{\lambda }$ is the spectral absorption coefficient of soot particles. Since the size of soot particles in the flame satisfies Rayleigh’s assumption, its absorption coefficient can be approximated by Mie theory as Equation (3).

(3) $$\kappa_{\lambda }\left(l\right)=\frac{6\pi E\left(m\right)f_{v}\left(l\right)}{\lambda }$$

where *f_v_*(*l*) is the volumetric concentration of soot in demand, *E*(*m*) is a function of the optical constant of soot, and *E*(*m*) is a function of the refractive index with real and imaginary parts. The value of *E*(*m*) is related to the physical properties of the soot particles. In this paper, *E*(*m*) is assumed to be 0.26 in the visible range, and *m* is 1.57–0.56*i*. Equation (2) is the flame radiative transfer equation without considering the scattering from the medium, from which the flame temperature field and the distribution of the soot concentration can be solved simultaneously. A CCD camera only captures the radiation intensity located in the range of its response wavelength, and then the *j*-line of sight of the camera can receive the red, green, and blue radiation energy from the flame *E_R_*(*j*), *E_G_*(*j*), and *E_B_*(*j*) as follows:

(4) $$\left\{\begin{array}{l} E_{R}\left(j\right)=\int_{\lambda_{aR}}^{\lambda_{bR}}\varphi_{\lambda ,R}\cdot I_{\lambda }\left(j\right)d\lambda =\int_{l_{0}\left(j\right)}^{l_{1}\left(j\right)}\int_{\lambda_{aR}}^{\lambda_{bR}}\varphi_{\lambda ,R}\cdot \kappa_{\lambda }\left(l\right)I_{b\lambda }\left(l\right)\left[\exp\int_{l_{0}\left(j\right)}^{l_{1}\left(j\right)}-\kappa_{\lambda }\left(l^{\prime }\right)dl^{\prime }\right]d\lambda dl\\ E_{G}\left(j\right)=\int_{\lambda_{aG}}^{\lambda_{bG}}\varphi_{\lambda ,G}\cdot I_{\lambda }\left(j\right)d\lambda =\int_{l_{0}\left(j\right)}^{l_{1}\left(j\right)}\int_{\lambda_{aG}}^{\lambda_{bG}}\varphi_{\lambda ,G}\cdot \kappa_{\lambda }\left(l\right)I_{b\lambda }\left(l\right)\left[\exp\int_{l_{0}\left(j\right)}^{l_{1}\left(j\right)}-\kappa_{\lambda }\left(l^{\prime }\right)dl^{\prime }\right]d\lambda dl\\ E_{B}\left(j\right)=\int_{\lambda_{aB}}^{\lambda_{bB}}\varphi_{\lambda ,G}\cdot I_{\lambda }\left(j\right)d\lambda =\int_{l_{0}\left(j\right)}^{l_{1}\left(j\right)}\int_{\lambda_{aB}}^{\lambda_{bB}}\varphi_{\lambda ,B}\cdot \kappa_{\lambda }\left(l\right)I_{b\lambda }\left(l\right)\left[\exp\int_{l_{0}\left(j\right)}^{l_{1}\left(j\right)}-\kappa_{\lambda }\left(l^{\prime }\right)dl^{\prime }\right]d\lambda dl \end{array}\right.$$

where $\exp\int_{l_{0}\left(j\right)}^{l_{1}\left(j\right)}-\kappa_{\lambda }\left(l^{\prime }\right)dl^{\prime }$ is the self-absorption term, which means part of the combustion radiation absorbed by the flame itself [25]. The self-absorption term makes it very difficult to solve, so it is often ignored in the process of solving. Equation (4) can be expressed in the following form:

(5) $$\left\{\begin{array}{l} E_{R}\left(j\right)=\int_{l_{0}\left(j\right)}^{l_{1}\left(j\right)}\int_{\lambda_{aR}}^{\lambda_{bR}}\varphi_{\lambda ,R}\kappa_{\lambda }\left(l\right)I_{b\lambda }\left(l\right)d\lambda dl=\int_{l_{0}\left(j\right)}^{l_{1}\left(j\right)}H_{R}\left(l\right)dl\\ E_{G}\left(j\right)=\int_{l_{0}\left(j\right)}^{l_{1}\left(j\right)}\int_{\lambda_{aG}}^{\lambda_{bG}}\varphi_{\lambda ,G}\kappa_{\lambda }\left(l\right)I_{b\lambda }\left(l\right)d\lambda dl=\int_{l_{0}\left(j\right)}^{l_{1}\left(j\right)}H_{G}\left(l\right)dl\\ E_{B}\left(j\right)=\int_{l_{0}\left(j\right)}^{l_{1}\left(j\right)}\int_{\lambda_{aB}}^{\lambda_{bB}}\varphi_{\lambda ,B}\kappa_{\lambda }\left(l\right)I_{b\lambda }\left(l\right)d\lambda dl=\int_{l_{0}\left(j\right)}^{l_{1}\left(j\right)}H_{B}\left(l\right)dl \end{array}\right.$$

where $\varphi_{\lambda }$ is the spectral response curve of the camera that can be obtained by calibration. Then, the only unknown quantities in Equation (5) are *T* and the spectral absorption coefficient $\kappa_{\lambda }(l)$. *H_R_*(*l*), *H_G_*(*l*), and *H_B_*(*l*) are the radiation source terms. According to the two-color method, *T* can be obtained by solving the ratio of flame spectral radiation intensity under two colors, *R* and *G* [26]:

(6) $$\frac{H_{R}(l)}{H_{G}(l)}=\frac{\int_{aR}^{bR}\varphi_{\lambda ,R}\frac{C_{1}}{\lambda^{6}(e^{C_{2}/(\lambda T(l))}-1)}d\lambda }{\int_{aG}^{bG}\varphi_{\lambda ,G}\frac{C_{1}}{\lambda^{6}(e^{C_{2}/(\lambda T(l))}-1)}d\lambda }$$

It is worth noting that the *T* obtained above is solved by ignoring the self-absorption term. When applying ES to small-scale flames, the self-absorption term is neglected as it has little effect on the calculation results. However, ignoring the self-absorption term for large-scale flames can cause significant interference in the calculation results. Therefore, it is necessary to include the effect of the self-absorption term [27,28]. By substituting the *T* obtained above into Equation (5), new radiation source terms *H_R_*(*l*) and *H_G_*(*l*) can be obtained. The flame temperature *T* is then recalculated based on the radiation source term, and the process is repeated until Equation (5) converges to obtain the final value of *T*. However, the consideration of self-absorption makes the calculation more complex and time-consuming. In addition, the multi-peak nature of the function makes it difficult to obtain an accurate solution. Therefore, developing a radiation equation to solve the multi-peak problem is particularly important for the calculation process. 

The PSO algorithm, based on the food-hunting behaviors associated with bird flocking, can deal with such problems [29,30]. The bird can be abstracted to a soot particle (NOx, etc.) without mass and volume, with its motion space extended to a *D*-dimensional space. Every PSO swarm is a solution in the solution space, which adjusts its flight according to its own and its companion’s flying experience. The best position in the course of the flight of each swarm is the best solution that is found by the swarm. The best position of the whole flock is the best solution, which is found by the flock. Every swarm continuously updates itself through the above-mentioned best solution. Thus, a new generation of community comes into being. The position of particle *i* is represented by the D-dimensional vector *X_i_*(*x*_1_, *x*_2_, …, *x_d_*). Each particle has a “fitness value” *f_i_*, which is a parameter determined by the objective function. The above process can be summarized as

(7) $$\left\{\begin{array}{c} V_{id}^{k+1}=wV_{id}^{k}+c_{1}r_{1}\left(P_{id}^{k}-X_{id}^{k}\right)+c_{2}r_{2}\left(P_{gd}^{k}-X_{id}^{k}\right)\\ X_{id}^{k+1}=X_{id}^{k}+V_{id}^{k+1} \end{array}\right.$$

where *w_i_* is the inertia weight factor, *d* = 1, 2, …, *D*, *i* = 1, 2, …, *n*. The parameter *k* is the number of iterations, *V_id_* is the particle velocity, *P_id_* is the best advantage that the particle itself passes through, *X_id_* is the position of the particle, and *P_gd_* is the best advantage that the population passes through. *c*_1_ and *c*_2_ are acceleration factors, which are constants. *r*_1_ and *r*_2_ are random numbers with values in the range of [0,1] and are considered uncertainty factors in the calculation process.

The APSO algorithm introduces the concept of a disturbance factor *f*, which is determined by the sum of the distances between each particle and the other particles [31]. The distance factor *d_i_* is calculated as follows:

(8) $$d_{i}=\frac{1}{N-1}\sum_{j=1,j\neq i}^{N}\sum_{k=1}^{D}\left|x_{ik}-x_{jk}\right|$$

Let the distance factor of the particle with the highest fitness in the existing particle swarm be *d_g_*, and the minimum and maximum of the distance factor in the particle swarm be *d_min_* and *d_max_*, respectively. The distribution factor *f* of the particle swarm is determined by

(9) $$f=\frac{d_{g}-d_{min}}{d_{max}-d_{min}}$$

**Figure 5:**
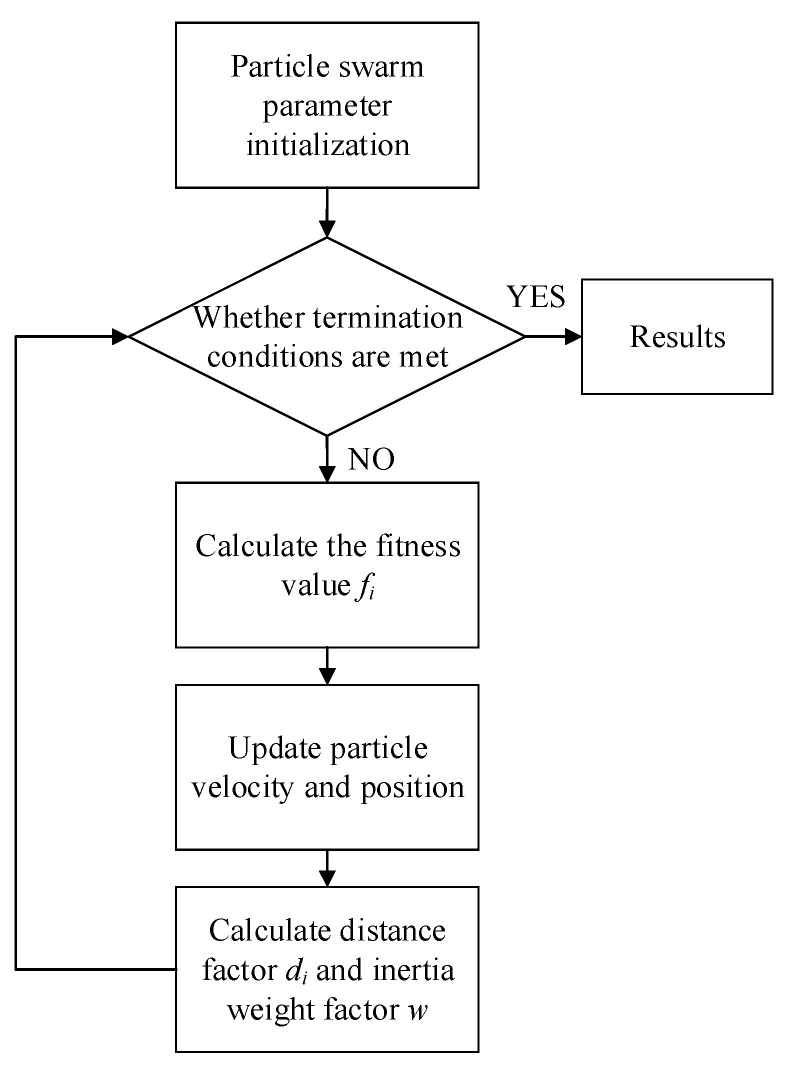
Flow chart of the APSO algorithm.

Then, the value of the inertia weighting factor *w_i_^k^*^+1^ is updated by

(10) $$w_{i}^{k+1}=f$$

where *f* = 1 means that the particle with the best position is farthest from other particles. By setting the inertia weight for the next iteration to *f*, the speed of other particles flying towards the optimal position can be reasonably controlled. When *f* takes a value in the range of [0,1], the closer the value of *f* approaches 1, the closer the value of the corresponding *w* in the next iteration to 1. The APSO enhances the global search ability of the particles and accelerates the speed of approaching the optimal particle. At the same time, a large number of particles are constantly in motion, resulting in an increasing computational complexity accordingly. The closer the value of *f* is to 0, the closer the optimal particle is to other particles, and the value of *w_i_* is also close to 0. As the local search ability of the particles is enhanced, the particles move less, which results in a corresponding decrease in computational complexity. Therefore, the APSO can reasonably control the speed of other particles flying towards the optimal position and adjust the computation complexity of the algorithm. As shown in Figure 5, the position and velocity of each particle are randomly initialized in the computational space. Then, the velocity and position of the particle are judged with the termination condition. If the conditions are not satisfied, then the fitness value *f_i_* of the particle will be calculated. If *f_i_* is better than the fitness of its own extreme *P_id_*, then the *P_id_* of that particle is updated to *X_id_*. If *f_i_* is better in the current iteration than the fitness of the global extreme *P_gd_*, the *P_gd_* is updated to *X_id_*. Finally, the distance factor *d_i_* and inertia weight factor *w_i_* are calculated.

In this study, a computer with an Intel (R) Core i7-6000T processor and a working frequency of 2.80 GHz was used for calculation. Five sets of experimental tests were conducted under different operating conditions to evaluate the calculation time of both the PSO- and APSO-optimized ES methods. When the population size and the iteration time were 100 and 5000, respectively, the computation time of the PSO-optimized ES was between 6.88 × 10^−8^ s and 11.67 × 10^−8^ s with an average of 9.74 × 10^−8^ s, while that of APSO was between 8.22 × 10^−8^ s and 9.94 × 10^−8^ s with an average of 9.06 × 10^−8^ s, indicating that the convergence time is less than that of the PSO. Table 1 shows the detailed calculation time of the two methods.

## 3. Experimental Results

### 3.1. Experimental Setup and Test Conditions

**Figure 6:**
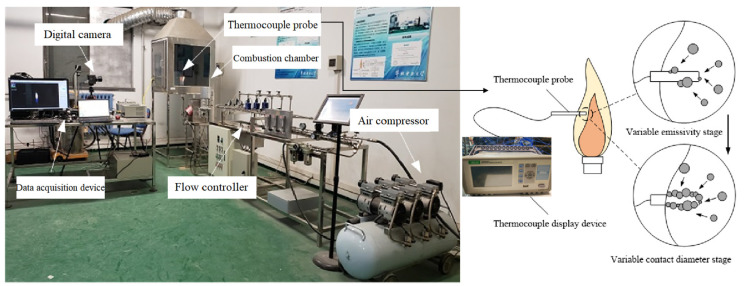
Test rig of diffusion methane flame.

Experimental tests were carried out on a gas-fired combustion rig, as shown in Figure 6. All experiments were conducted on diffusion flames by burning methane at volumetric flow rates of 0.6 to 0.9 L/min. For a diffusion flame, the change in the fuel flow rate leads to the variation in the jet velocity of the fuel and, thus, affects the rate of chemical reactions and buoyancy convection. The fuel flow rate was measured by a float flow meter and regulated by the flow controller. A burner with an outer diameter of 24 mm was adopted to form diffusion flames in a combustion chamber with a side length of 800 mm and a height of 700 mm. The flame images were obtained using a digital camera (Photron, FASTCAM Mini UX50, PHOTRON USA, Inc., San Diego, CA, USA), which acquires the flame images at a rate of 250 frames per second and with a resolution of 1280 × 1024 pixels.

**Figure 7:**
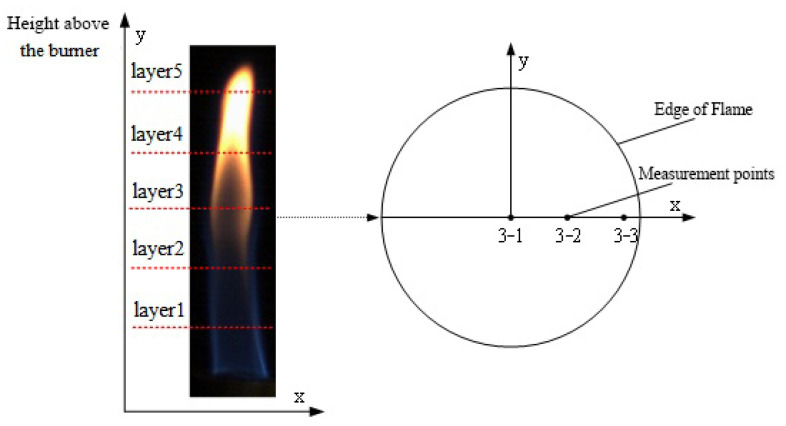
Distribution of flame measurement points.

In order to compare the results with those of ES and IES, the TPD method was used to obtain soot concentrations under different operating conditions along five height layer measurement points, as shown in Figure 7. The soot concentration was obtained in different height layers of the flame, with each layer taking the center, the mid-point of the radius, and the edge of the flame as the measurement points. Each point is numbered in the X–Y form, where X is the height layer of the flame and Y is the radial point number. The measurements were repeated three times at each measurement point and the average was used for analysis. Before each measurement, the soot particles deposited on the surface of the thermocouple must be removed. The output voltage from the thermocouple (with a diameter of 1 mm) was sampled at 50 Hz for 10 s.

### 3.2. Reconstruction of Soot Concentration

**Figure 8:**
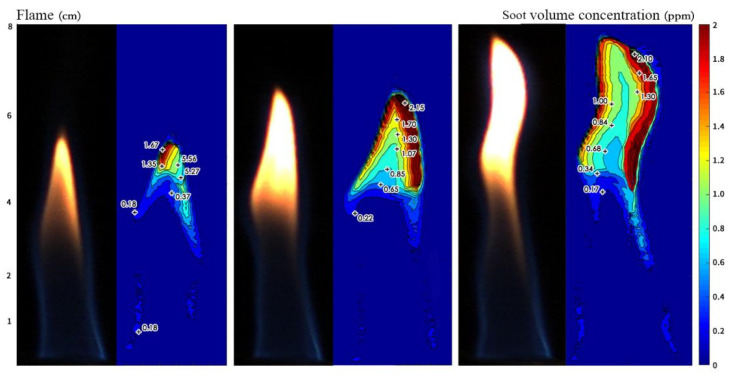
Original image of flame and soot distribution at methane flowrate of 0.7 L/min.

The original image of the flame with the soot concentration is shown in Figure 8. The flame’s soot content is approximately 2 ppm (parts per million, 1 ppm = 1 ug/g). Compared to the reference available [32], it is presumably since the combustion in the reference was carried out in a large combustion furnace with a high chamber temperature and a flame length about ten times longer than in the present experimental conditions.

**Figure 9:**
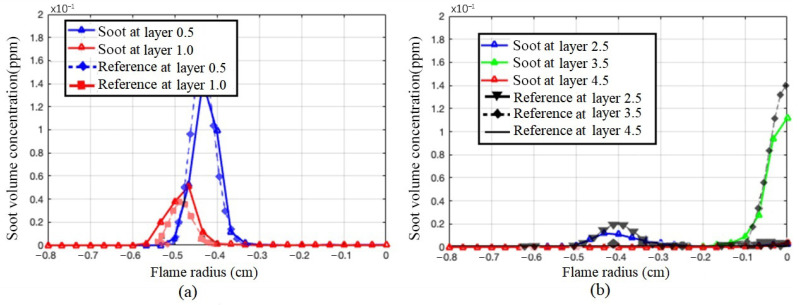
Images of soot concentration at (**a**) layer 0.5 and 1.0 and (**b**) layer 2.5, 3.5 and 4.5 of the flame.

The measured soot concentrations of a methane/air diffusion flame under the same test condition of reference [33] are shown in Figure 9. When the detection height is less than 3/4 of the overall flame height, the soot concentration has a peak close to the flame center. With the increase in flame height, the concentration varies between 60 and 120 ppm. At a height higher than 3/4 of the flame, the soot concentration gradually increases as the position is close to the flame center. In layer 3.5, the soot concentration is at a high level. The soot concentration rises gradually as the position moves closer to the flame center. This is probably because layer 3.5 is the brightest part of the flame, so the soot content is the highest. In layer 4.5, which is the top part of the flame, the soot content starts to decrease and maintains a gradual increase trend near the flame center. The maximum value near the flame center is 0.0404 ppm, which is ten times higher than that at the root of the flame. 

**Figure 10:**
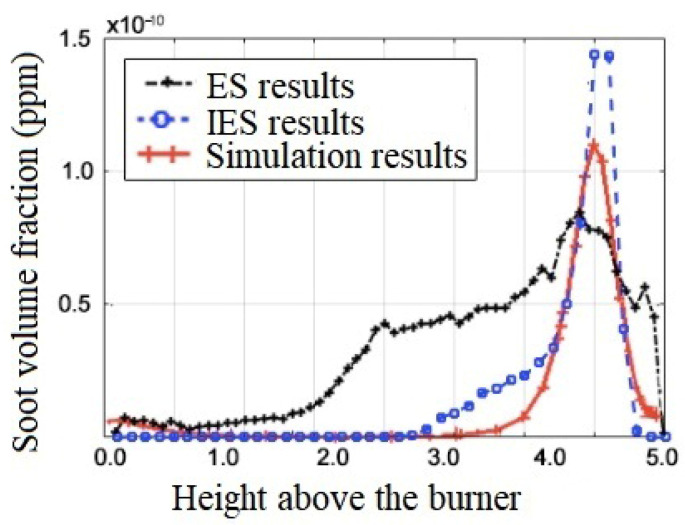
Comparison of two methods and simulated values of soot volume fraction.

The soot concentration obtained by ES and IES methods was compared with those from Chemkin simulation [34] under the methane flowrate of 0.7 L/min. Since the simulation results are derived from one-dimensional flames, where each data point is an average of all values at that flame height, the experimental values were also processed in the same way. As can be seen from Figure 10, the soot concentration increases gradually with the flame height up to layer 4 and then decreases gradually. This may be due to the fact that the content of fuel gases involved in the combustion reaction decreases dramatically in the top part of the flame. It can also be seen from Figure 10 that the simulation results and the measured results of the IES method are in good agreement, while a significant difference is observable compared to those of the ES method.

In addition, at the flame height of layer 4.5, the volume fraction of soot obtained by the three methods presents an obvious unimodal distribution, and the peak value measured by the IES method is higher than that measured by the ES method and simulation results. This may be because the IES method considers the self-absorption term of soot radiation in its calculation, while the other two methods do not take it into account.

**Figure 11:**
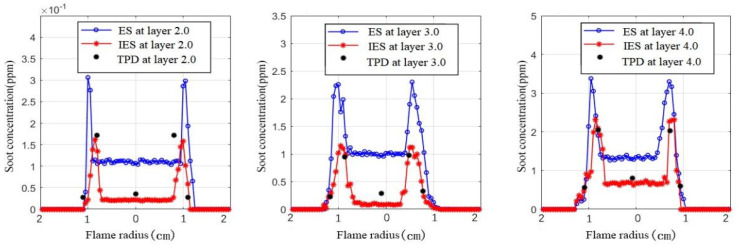
Comparison of different methods of measuring the soot concentration at different heights of the flame.

In order to further demonstrate the improvement in the IES method, the results obtained by ES and IES methods were compared with those of the TPD method under the methane flowrate of 0.7 L/min. As shown in Figure 11, the soot concentration in each height layer of the flame varies along the flame radius with a saddle shape. On the edge of the flame, the amount of fuel gas involved in the combustion reaction gradually decreases, while the amount of exposed outside air increases, resulting in a gradual decrease in the concentration of soot particles due to oxidation reactions. Near the edge of the flame, the equivalent air-to-fuel ratio and temperature are higher than that of the outer flame because the formation of soot is a heat-absorbing reaction. There is an oxidation transition zone between the inner flame’s soot formation zone and the outermost flame’s soot oxidation zone. Moreover, at different heights of the flame, the soot concentration measured by the IES method is more consistent with that of the TPD method compared to the ES method. The soot concentration measured by ES, IES, and TPD methods and the corresponding standard deviation are provided in Table 2. Experimental tests were conducted with a methane flow rate of 0.7 L/min on measurement layer 4. As can be seen from Table 2, compared to the TPD algorithm, the ES method has a maximum absolute deviation of 0.84 ppm from the average soot concentration at the three measurement points, while that of the IES is only 0.09 ppm, indicating that the IES method has better repeatability. Moreover, the standard deviations of the IES method are generally lower than those of the TPD method. This shows that the data obtained by the IES is more concentrated and less fluctuating.

It can be seen from the measurement results that the two-dimensional distribution of flame soot concentration calculated by the IES method can more accurately reflect the actual situation of the flame, which is also in good agreement with the measurement results of the literature and the TPD method. Therefore, the proposed algorithm can predict the distribution of soot concentration in small-size diffusion flames and help understand the reaction mechanism in combustion. 

## 4. Conclusions

The proposed IES method is capable of measuring the soot concentration of diffusion flames under different operating conditions with better performance than the conventional ES method. The embedded APSO algorithm was used to handle the multi-peak function optimization problems of the ES method. The two-dimensional reconstruction of the soot concentration of the flame was realized, and it was found from the results that the soot particles from incomplete combustion appeared in the upper part of the flame. Compared to the TPD algorithm, the IES method had a maximum absolute deviation of only 0.09 ppm, which is much lower than that of the ES method.

## Figures and Tables

**Table 1:** Comparison of calculation time between the PSO and the APSO algorithm (×10^−8^ s).

Algorithms	1	2	3	4	5	Average
PSO	11.56	11.67	8.73	6.88	9.88	9.74
APSO	9.94	8.22	8.48	9.39	9.29	9.06

**Table 2:** Comparison of the averaged soot concentration and the standard deviation at three measurement points in layer 4 (methane flow rate: 0.7 L/min).

Methods	Average Soot Concentration (ppm)			Standard Deviation (ppm)		
	Point 4–1	Point 4–2	Point 4–3	Point 4–1	Point 4–2	Point 4–3
TPD	0.85	2.18	0.64	0.29	0.67	0.074
ES	1.42	3.02	0.60	0.08	0.057	0.042
IES	0.76	2.25	0.70	0.04	0.063	0.051

## Data Availability

All the data supporting reported results can be obtained by contacting the corresponding author through email.
